# Leaf Area Index Drives Soil Water Availability and Extreme Drought-Related Mortality under Elevated CO_2_ in a Temperate Grassland Model System

**DOI:** 10.1371/journal.pone.0091046

**Published:** 2014-03-14

**Authors:** Anthony Manea, Michelle R. Leishman

**Affiliations:** Department of Biological Sciences, Macquarie University, North Ryde, NSW, Australia; University of Antwerp, Belgium

## Abstract

The magnitude and frequency of climatic extremes, such as drought, are predicted to increase under future climate change conditions. However, little is known about how other factors such as CO_2_ concentration will modify plant community responses to these extreme climatic events, even though such modifications are highly likely. We asked whether the response of grasslands to repeat extreme drought events is modified by elevated CO_2_, and if so, what are the underlying mechanisms? We grew grassland mesocosms consisting of 10 co-occurring grass species common to the Cumberland Plain Woodland of western Sydney under ambient and elevated CO_2_ and subjected them to repeated extreme drought treatments. The 10 species included a mix of C_3_, C_4_, native and exotic species. We hypothesized that a reduction in the stomatal conductance of the grasses under elevated CO_2_ would be offset by increases in the leaf area index thus the retention of soil water and the consequent vulnerability of the grasses to extreme drought would not differ between the CO_2_ treatments. Our results did not support this hypothesis: soil water content was significantly lower in the mesocosms grown under elevated CO_2_ and extreme drought-related mortality of the grasses was greater. The C_4_ and native grasses had significantly higher leaf area index under elevated CO_2_ levels. This offset the reduction in the stomatal conductance of the exotic grasses as well as increased rainfall interception, resulting in reduced soil water content in the elevated CO_2_ mesocosms. Our results suggest that projected increases in net primary productivity globally of grasslands in a high CO_2_ world may be limited by reduced soil water availability in the future.

## Introduction

A major driver that shapes the physiology, ecology and evolution of terrestrial plants is climatic extremes [1]. It is widely acknowledged that the magnitude and frequency of climatic extremes, such as drought, are likely to increase under future climate change conditions [2]. The IPCC [2] defines a climatic extreme, such as extreme drought, as an event that occurs once every 20 years, on average. The potential for climatic extremes to alter the structural and functional dynamics of ecological communities [2], [3], coupled with their increasing magnitude and frequency in the future, suggests research on climatic extremes should be a high priority.

Grass-dominated systems (grasslands, savannas and open grassy woodlands) occupy more than 30% of the global terrestrial landscape [4] and play an important role in the global carbon cycle [5]. The productivity of grass-dominated systems (referred to as grasslands from now on) is strongly mediated by soil water availability [4], [6], [7]. For example, Fay et al [8] increased the mean annual rainfall to an experimental grassland community in Kansas by 250% and found that both soil water content (SWC) and aboveground net primary productivity significantly increased (see exception [9]). Consequently it is likely that soil water availability prior to an extreme drought event will be a major driver in the response of the grassland to the event.

It has rarely been considered how CO_2_ concentration will alter soil water availability in grasslands and thus modify grassland responses to extreme drought. One of the major drivers of soil water availability in grasslands is canopy transpiration [6]. The amount of water that is lost through canopy transpiration depends on the stomatal conductance and leaf area index (LAI) of the grasses [10]. Reduction in stomatal conductance of grasses under elevated CO_2_ has been well-documented [6], [11], [12]. For example, Morgan et al [7] found that in the Wyoming mixed-grass prairies in the United States, the annual SWC increased on average by 17.3% over a three year period due partly to reductions in stomatal conductance under elevated CO_2_ levels. The physiology (C_3_ or C_4_) and origin (native or exotic) of the grasses play an important role in this reduction of stomatal conductance under elevated CO_2_ levels. C_4_ plants evolved in a low CO_2_ environment, allowing high rates of photosynthesis at low stomatal conductance [13]. This suggests that C_4_ plants should have greater reductions in stomatal conductance compared to C_3_ plants under elevated CO_2_ levels. In addition, meta-analysis studies have shown that at the leaf-level natives tend to have lower stomatal conductance than exotics [14].

In contrast to reductions in stomatal conductance, grasses often have higher leaf area index (LAI) under elevated compared to ambient CO_2_ levels, resulting in increased canopy transpiration. For example, the LAI of the C_4_ bunchgrass *Pleuraphis rigida* significantly increased under elevated CO_2_ in the 10 year Mojave Desert FACE experiment [15]. In a meta-analysis of semi-wild and wild C_3_ and C_4_ grasses, leaf area (related to LAI) increased under elevated CO_2_ by 15% and 25% respectively [16]. Therefore reductions in the stomatal conductance of grasses under elevated CO_2_ may be offset by increases in their LAI [17].

In this study we asked whether the response of grasslands to repeat extreme drought events is modified by elevated CO_2_, and if so, what are the underlying mechanisms? Experimental mesocosms containing common co-occurring native and exotic grass species of the Cumberland Plain Woodland of western Sydney, Australia were grown under ambient and elevated CO_2_ levels and exposed to repeated one in 20 year extreme drought events. We hypothesized that a reduction in the stomatal conductance of the grasses, particularly the C_4_ and native grasses, grown under elevated CO_2_ levels would be offset by increases in the LAI of the grasses. That is, the total canopy transpiration of the grasses would not differ between the CO_2_ treatments. Therefore the retention of soil water in the mesocosms and consequently the vulnerability of the grasses to extreme drought would not differ between the CO_2_ treatments.

## Methods

### Species selection

We selected five native and five exotic perennial grass species which commonly co-occur in a grassy open woodland community known as Cumberland Plain Woodland that occurs in western Sydney, Australia. All the exotic species are considered to be invasive rather than simply exotics that have become naturalized in Cumberland Plain Woodland [18]. Within both the native and exotic groups we included two C_3_ and three C_4_ species. Seeds for the 10 grass species were obtained from a commercial supplier (Nindethana Seed Service, Albany, WA, Australia). Information on the biology of each grass species is provided in table 1. Once collected, the seeds for each of the 10 grass species were germinated on moist paper towels within covered aluminium trays. To spread the risk of germination failure, each grass species was germinated in a number of different aluminium trays.

**Table 1:** Grass species used in the study.

Species	Origin	Seed mass (mg)	Photosynthetic pathway	Longevity
*Chloris gayana* Kunth	Exotic	0.4	C_4_	Perennial
*Eragostis curvula* (Schrad.) Nees	Exotic	0.2	C_4_	Perennial
*Pennisetum clandestinum* Hochst. Ex Chiov	Exotic	7.0	C_4_	Perennial
*Bromus catharticus* Vahl	Exotic	10.7	C_3_	Short lived perennial
*Ehrharta erecta* Lam.	Exotic	2.0	C_3_	Perennial
*Bothriochloa macra* (Steud.) S.T.Blake	Native	1.2	C_4_	Perennial
*Chloris truncata* R.Br.	Native	0.3	C_4_	Perennial
*Themeda australis* (R.Br.) Stapf	Native	2.6	C_4_	Perennial
*Austrodanthonia racemosa* (R.Br.) H.P.Linder	Native	0.7	C_3_	Perennial
*Microlaena stipoides* (Labill.) R.Br.	Native	4.3	C_3_	Perennial

Grass species (family: *Poaceae*) used in this study, with information on the origin, seed mass, photosynthetic pathway and longevity of each species. Average seed mass was obtained by oven drying 50 seeds from each species at 60°C for 48 hours and then weighing them. Data on taxonomy, physiology and longevity were obtained from PlantNET (www.plantnet.rbgsyd.nsw.gov.au).

### Experimental design and treatments

The native and exotic grass species were grown together in mesocosms using a fully factorial experimental design with two factors: CO_2_ concentration and drought treatment. The mesocosms consisted of 65 L tubs (60 cm long ×40 cm wide ×28 cm deep), each tub containing 55 L of soil mixture consisting of field-collected Cumberland Plain Woodland soil and coarse river sand in a ratio of 3∶1. The Cumberland Plain Woodland soil was obtained from Mt Annan (34.07°S, 150.76°E) and Luddenham (33.88°S, 150.69°E) in western Sydney and was homogenized into a single batch. No specific approvals or permits were required for soil collection. The soil collection locations were not privately-owned or protected in any way and did not endanger any protected species. The river sand was obtained from a commercial supplier (Australian Native Landscapes, North Ryde, NSW, Australia). Seedlings were transplanted from the germination trays into the CO_2_ treatment mesocosms at the stage of second true leaf emergence. All seedlings were planted within 24 hours of each other. The seedlings were planted in two rows of five with each species allocated a position randomly within each mesocosm. For each grass species multiple seedlings were transplanted into each mesocosm as insurance against seedling mortality. After six days, the excess seedlings were removed from the mesocosms, leaving one individual per species per mesocosm.

CO_2_ treatments were set to two levels: ambient (380–420 ppm) and elevated (530–570 ppm) CO_2_. These CO_2_ concentration ranges were maintained and monitored daily by a CO_2_ dosing and monitoring system (Canary Company Pty Ltd, Lane Cove, NSW, Australia). The lower concentrations of these ranges tended to occur at night-time while the higher concentrations occurred during the daytime. The elevated CO_2_ treatment represents the predicted atmospheric CO_2_ concentration by 2050 [19].

We defined a drought as the number of consecutive days with <1 mm of rainfall. This definition is a part of the ETCCDI/CRD climate change indices [20]. We used the IPCC [2] definition of climatic extreme which is an extreme that occurs once every 20 years, on average. Gumbel I distributions were fitted to the annual drought extremes of the Cumberland Plain for each year from 1867–2010. Rainfall on the Cumberland Plain is not seasonal so the time of year that the annual drought extremes occurred differed from year to year. The data used were obtained from the Australian Bureau of Meteorology historical records of Brownlow Hill (34.03°S, 150.65°E, 1867–1969), Kentlyn (34.05°S, 155.88°E; 1970–1971), Camden Airport (34.04°S, 150.69°E; 1972–1992, 1998–2001), Ruse (34.06°S, 150.85°E; 1993–1997) and Mt Annan Botanical Gardens (34.07°S, 150.76°S; 2002–2010). A one in 20 year extreme drought event for the Cumberland Plain was calculated to last for a period of 53 days. The extreme drought was simulated by turning off the watering system for the treatment period.

The extreme drought treatment mesocosms were replicated five times at each CO_2_ level. These were called the ‘drought treatment’ mesocosms. In addition, five extra mesocosms were grown under each CO_2_ level. These were called the ‘before treatment’ mesocosms. All mesocosms were mist watered for one minute twice daily which is representative of the average daily amount of rainfall (828 mm annually) on the Cumberland Plain. This daily rainfall average was based on the same Australian Bureau of Meteorology historical records from Camden airport (1943–2004) as described in the above paragraph. This design resulted in a total of 20 mesocosms each containing 10 grass species (i.e. [2 CO_2_ treatments ×5 replicates]+10 extra mesocosms). The mesocosms within each CO_2_ treatment were split between two glasshouses. The mesocosms were switched between glasshouses within each CO_2_ treatment once during the growth period and once during the treatment period to reduce any glasshouse effect. The temperature of the glasshouses was set for a maximum of 24°C and a minimum of 16°C. During the entire duration of the experiment the relative humidity within each glasshouse was measured every day at 9am and 3pm using a HOBO temperature/RH/2 external channel data logger (OneTemp, Parramatta, NSW, Australia).

The grasses were grown for 12 weeks at which stage they were mature and seeding. After the growth period the ‘before treatment’ mesocosms were harvested, washed free of soil and separated into their following components: leaf biomass, stem biomass and belowground biomass. The total leaf area of the leaf biomass was measured using a LI-3100C Area Meter (Li-Cor, Lincoln, NE, United States). The plant components were then oven-dried at 60°C for 72 hours and weighed using a Mettler Toledo B-S electronic balance.

The extreme drought treatment was then applied to the five ‘drought treatment’ mesocosms at each CO_2_ level. After the 53 day treatment period the mesocosms were mist-watered twice daily as previously for four weeks to allow a recovery period. In field conditions on the Cumberland Plain four weeks would be ample time for the grasses to recover (i.e. resprout) from a dry period. For 30 days prior to and during the drought/recovery cycles the SWC of each mesocosm was measured at a depth of 15 cm every 10 days using a Hydrosense II Portable Soil Moisture System (Campbell Scientific Australia Pty Ltd, Garbutt, QLD, Australia). In addition, for the 30 days prior to the extreme drought treatment the stomatal conductance of each grass was measured every five days using a Porometer AP4 (Delta-T Devices, Burwell, CB, Uniting Kingdom). Measurements would begin at 8.30 am and would take approximately three and a half hours to complete. After the four week recovery period, the mortality of the grasses was recorded. Each grass was classified as dead or alive depending on if it showed signs of regeneration by the end of the recovery period. The grasses were then exposed to two more cycles of drought and recovery with mortality of the grasses recorded after each cycle. At the end of the recovery period of the final cycle, every mesocosm was dug up to ensure that the grasses classified as dead had no living root material.

### Data analysis

#### Leaf area index and biomass allocation analysis

We calculated LAI and root to shoot ratio (R∶S) based on the harvested biomass data of the ‘before treatment’ mesocosms. LAI for each individual grass was calculated as total leaf area divided by ground area in the mesocosm (0.24 m^2^). R∶S for each individual grass was calculated as total root mass divided by total shoot mass. We used two-way ANOVAs to test for a CO_2_ and species effect on LAI, R∶S and total biomass across all grass species in the ‘before treatment’ mesocosms.

We used two-way ANOVAs to test for a CO_2_ and plant type (physiology and origin) effect on LAI across all grass species in the ‘before treatment’ mesocosms.

#### CO2 level and plant type survival analysis

Kaplan-Meier survival curves were generated to determine the survival function across all the grass species in relation to (1) CO_2_ level, (2) physiology, (3) origin and (4) CO_2_ level combined with each plant type (origin and physiology). If there was a significant difference between the Kaplan-Meier survival curves within each CO_2_ level/plant type combination then the survival distributions within the combination were tested for a significant difference. All survival distributions were compared using log rank tests, with significance set at P<0.05.

#### Stomatal conductance, total canopy transpiration and soil water content analysis

We used General Estimating Equation (GEE) models with a Bonferroni adjustment to determine if there was a difference in the stomatal conductance between CO_2_ level and each plant type (origin and physiology) combination during the 30 days prior to the extreme drought treatment. The same analyses were performed for SWC with the addition of testing for a difference between the CO_2_ levels for the three drought/recovery cycles. For all GEE models we specified a Gamma with log-link model using an exchangeable correlation matrix for the continuous variables (stomatal conductance and SWC).

Total canopy transpiration for each species was calculated as the average stomatal conductance multiplied by the average LAI. We carried out species-pair comparisons using paired t-tests with a Bonferroni adjustment to test for a CO_2_ effect on total canopy transpiration.

#### Relative humidity analysis

We used a paired t-test with a Bonferroni adjustment to determine if there was difference in the mean daily relative humidity between the ambient and elevated CO_2_ glasshouses. The relative humidity data was then averaged within each CO_2_ level and compared to the historical average relative humidity (9am-74%, 3pm-49%) of the Cumberland Plain using one-sample t-tests. This involved separate analyses for the relative humidity data obtained at 9am and 3pm. The historical average used was obtained from the Australian Bureau of Meteorology historical records of Camden Airport (34.04°S, 150.69°E; 1943–2010).

All data analyses were performed using IBM SPSS statistical software, Version 21.0.0 (SPSS Inc., 2012, IBM, Illinois, United States, http://www.spss.com) with the significance level set at 0.05. Data were log_10_ transformed when necessary to fulfil the assumptions of ANOVA.

## Results

### Humidity analysis

There was no significant difference in the relative humidity between and ambient and elevated CO_2_ glasshouses (t_1,38_ = 1.00, p = 0.326). However the relative humidity in the glasshouses at 9am (t = 10.01, p<0.001) and 3pm (t = 17.78, p<0.001) was significantly higher than the historical average for the same times of day.

### Leaf area index and biomass allocation analysis

There was no significant interaction between CO_2_ and species for LAI, R∶S or total biomass but there was a significant difference between species for all traits. LAI (F_1,99_ = 14.20, p<0.001) and total biomass (F_1,99_ = 19.97, p<0.001) were significantly higher under elevated CO_2_ compared to ambient CO_2_ across all the grass species. R∶S (F_1,99_ = 0.26, p = 0.615) did not significantly differ between ambient and elevated CO_2_.

**Figure 1:**
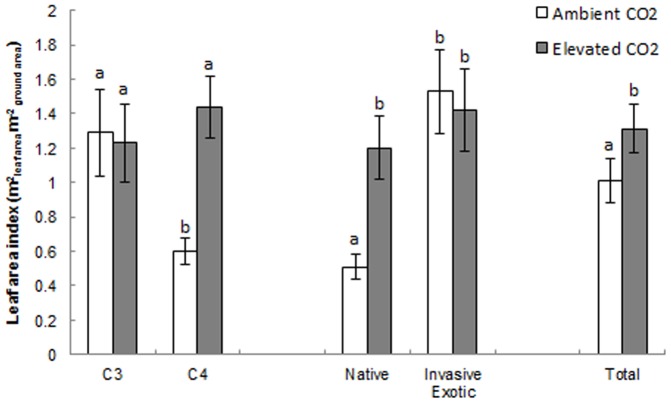
Leaf area index across all grass species for each CO_2_×plant type combination. Mean leaf area index across all grass species for each CO_2_×physiology and CO_2_×origin combination. Error bars represent one standard error. Letters indicate significant differences at p<0.05.

There was a significant interaction between CO_2_ and physiology for LAI (F_1,99_ = 5.15, p = 0.026) with the C_4_ grasses grown under ambient CO_2_ having significantly lower LAI than all of the other CO_2_×physiology combinations (figure 1). There was also a significant interaction between CO_2_ and origin for LAI (F_1,99_ = 4.21, p = 0.043) with the native grasses grown under ambient CO_2_ having significantly lower LAI than all of the other CO_2_×physiology combinations (figure 1).

### CO_2_ level and plant type survival analysis

The average survival rate across all grass species was significantly higher under ambient compared to elevated CO_2_ levels (χ^2^ = 8.58, df = 1, p = 0.003). The physiology (χ^2^ = 3.24, df = 1, p = 0.072) or origin (χ^2^ = 0.03, df = 1, p = 0.855) of the grasses did not significantly influence their survival rates.

**Figure 2:**
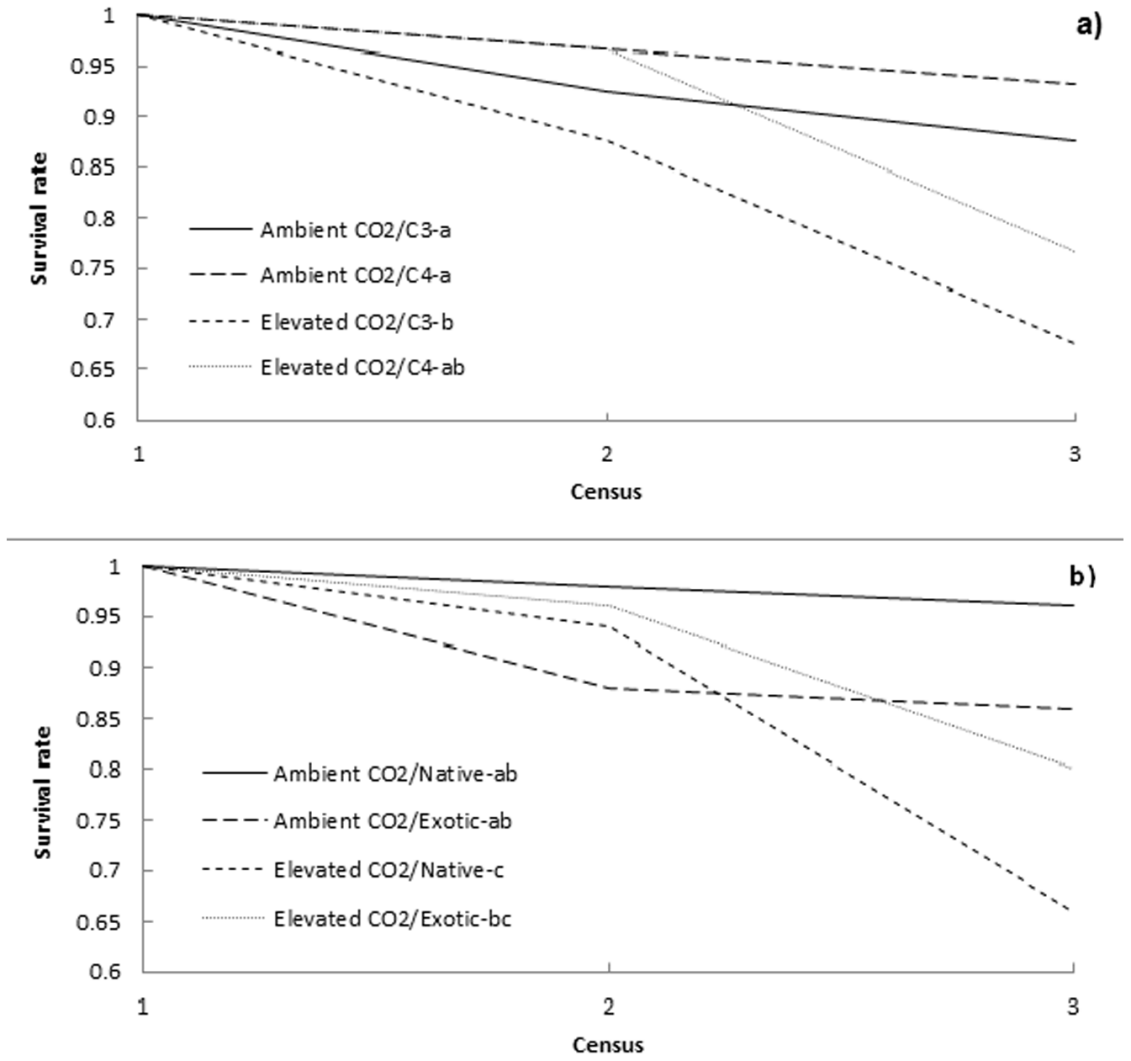
Survival rates across all grass species for each CO_2_×plant type combination. The average survival rate across all grass species of each (a) CO_2_×physiology and (b) CO_2_×origin combination, at each survival census. A survival census was carried out after each extreme drought cycle which consisted of a 53 extreme drought period and 28 day recovery period. Letters indicate significant differences at p<0.05.

There was a significant difference in survival between the different CO_2_×physiology combinations (χ^2^ = 13.29, df = 3, p = 0.004; figure 2a). Both the C_3_ (χ^2^ = 10.01, df = 1, p = 0.002) and C_4_ (χ^2^ = 7.91, df = 1, p = 0.005) grasses grown under ambient CO_2_ had significantly higher survival rates than the C_4_ grasses grown under elevated CO_2_.

There was a significant difference in survival between the different CO_2_×origin combinations (χ^2^ = 16.64, df = 3, p = 0.001; figure 2b). The native grasses grown under ambient CO_2_ had a significantly higher survival rate than the native (χ^2^ = 17.45, df = 1, p<0.001) and exotic (χ^2^ = 6.52, df = 1, p = 0.011) grasses grown under elevated CO_2_. The exotic grasses grown under ambient CO_2_ had a significantly higher survival rate than the native grasses grown under elevated CO_2_ (χ^2^ = 4.13, df = 1, p = 0.042).

### Stomatal conductance, total canopy transpiration and soil water content analysis

Stomatal conductance across all grass species was significantly lower under elevated CO_2_ compared to ambient CO_2_ (Wald χ^2^ = 7.254, df = 1, p<0.007).

**Figure 3:**
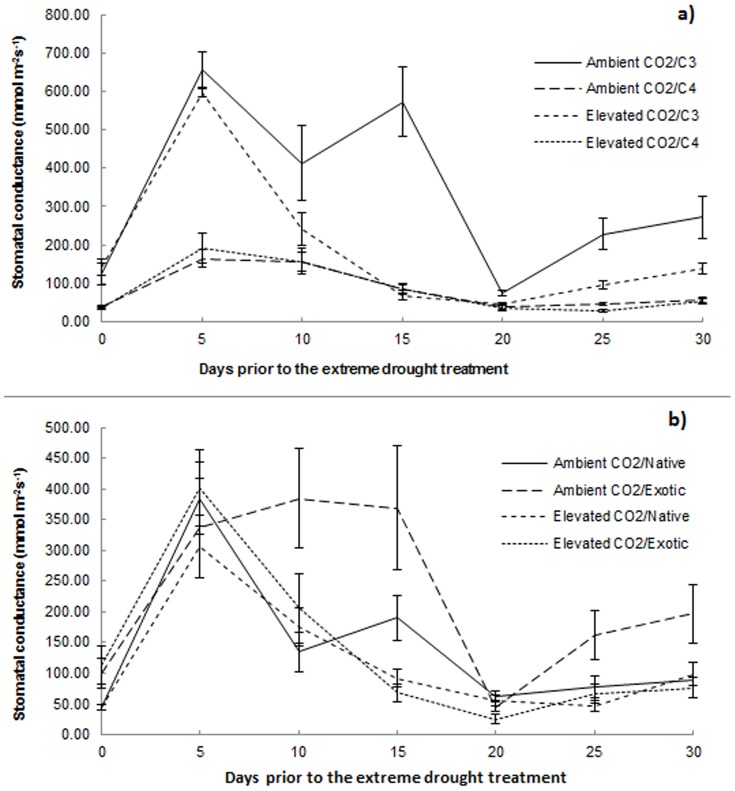
Stomatal conductance across all grass species for each CO_2_×plant type combination. Mean stomatal conductance across all grass species for each (a) CO_2_×physiology and (b) CO_2_×origin combination, during the 30 days prior to the extreme drought treatment. Error bars represent one standard error.

There was no significant interaction between CO_2_ and physiology for stomatal conductance (Wald χ^2^ = 1.786, df = 1, p = 0.181). The C_3_ grasses had significantly higher stomatal conductance than the C_4_ grasses (Wald χ^2^ = 46.00, df = 1, p<0.001; figure 3a). As described above, stomatal conductance was significantly lower under elevated CO_2_ compared to ambient CO_2_.

There was a significant interaction between CO_2_ and origin for stomatal conductance (Wald χ^2^ = 7.369, df = 1, p = 0.007; figure 3b). The exotics grown under ambient CO_2_ had significantly higher stomatal conductance than all of the other CO_2_×origin combinations.

There was no significant difference in the total canopy transpiration between ambient and elevated CO_2_ treatments (t_8_ = −0.93, p = 0.375).

**Figure 4:**
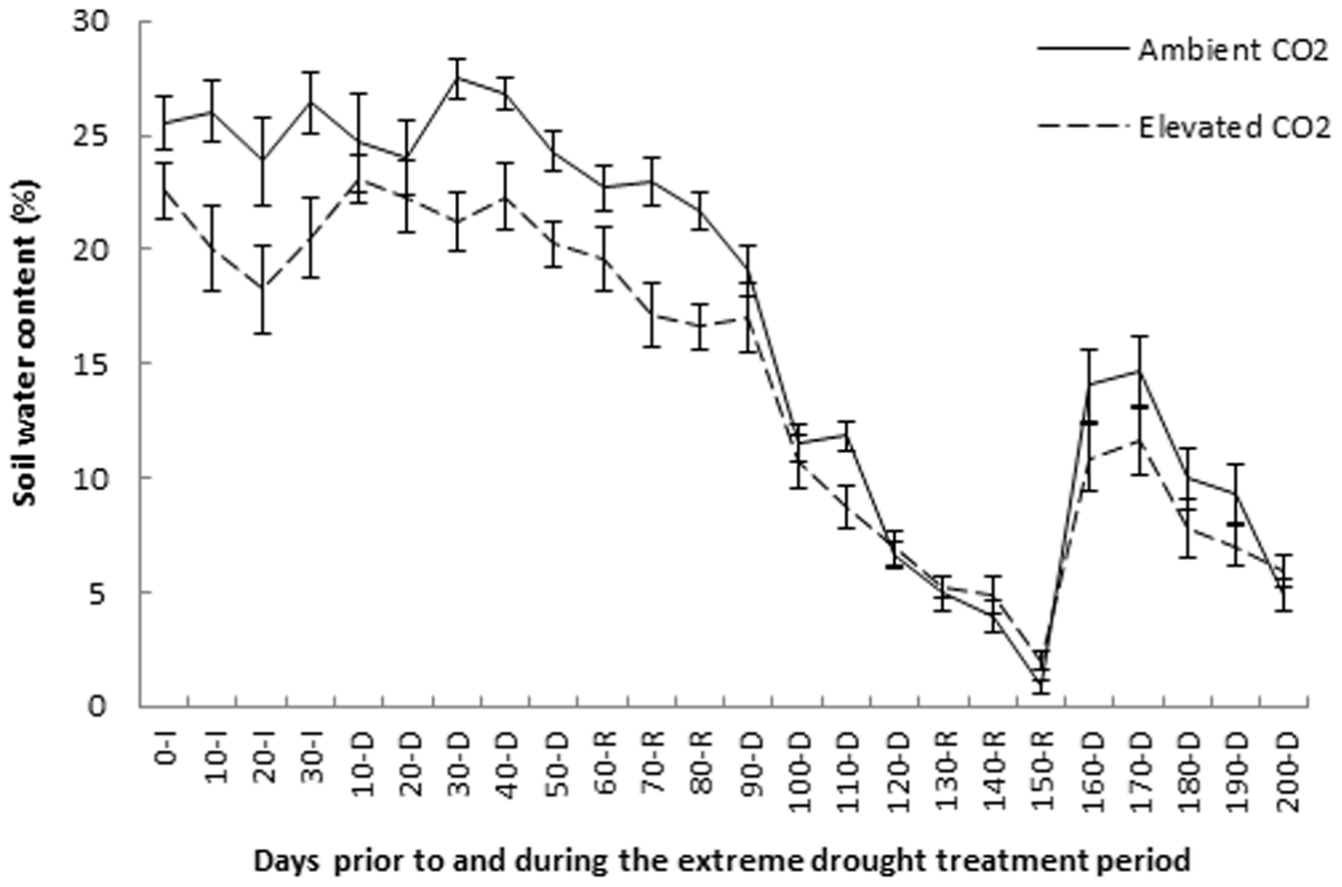
Soil water content across all mesocosms under ambient and elevated CO_2_ levels. Mean soil water content across all mesocosms under ambient and elevated CO_2_ levels over the 30 days prior to the extreme drought treatment and the drought/recovery cycles. The letters on the x-axis represent different phases of the experiment with I = the 30 day initial period prior to the extreme drought treatment, D = drought period and R = recovery period. Error bars represent one standard error.

The SWC during the 30 days prior to the drought/recovery cycles was significantly higher under ambient CO_2_ compared to elevated CO_2_ (Wald χ^2^ = 5.91, df = 1, p = 0.015). The overall SWC of the mesocosms during the experiment drought/recovery cycles was significantly higher under ambient CO_2_ compared to elevated CO_2_ (Wald χ^2^ = 11.48, df = 1, p = 0.001; figure 4). However SWC differences varied between drought/recovery cycles. During the first drought/recovery cycle SWC was significantly higher in the ambient compared with elevated CO_2_ treatment (Wald χ^2^ = 8.98, df = 1, p = 0.003). During the second and third drought/recovery cycles SWC did not significantly differ between CO_2_ treatments (second cycle Wald χ^2^ = 0.71, df = 1, p = 0.400; third cycle Wald χ^2^ = 2.504, df = 1, p = 0.114).

## Discussion

In this study we tested the vulnerability of an experimental grassland community to repeat extreme drought events under ambient and elevated CO_2_ levels. Our results show that grasses grown under elevated CO_2_ had significantly higher mortality in response to extreme drought than those grown under ambient CO_2_. Our original hypothesis was that SWC of the mesocosms and thus vulnerability of the grasses to extreme drought would not differ between the CO_2_ treatments because the reductions in stomatal conductance would be offset by increases in LAI. Our results did not support this hypothesis: soil water content was significantly lower in the elevated CO_2_ treatment thus increasing extreme drought-related mortality in the grasses. The 32% reduction in stomatal conductance under elevated CO_2_ was offset by a 30% increase in LAI. This offset is shown by the non-significant difference in total canopy transpiration of the grasses between the CO_2_ treatments. These results contrast the results of previous studies that have found that the SWC in semi-arid grasslands increased under elevated CO_2_ because the increases in total leaf area (related to LAI) were insufficient to offset the decreases in stomatal conductance [7], [21].

As the amount of water lost through total canopy transpiration did not differ between the CO_2_ treatments, differences in SWC must have been due to the amount of water reaching the soil surface. Rain throughfall decreases with LAI because of increased interception and subsequent evaporation of rainfall from the surfaces of leaves [10]. In this experiment the grasses had significantly higher LAI under elevated CO_2_. This would have increased rainfall interception consequently causing differences in the SWC between the CO_2_ treatments prior to the extreme drought treatments. As the experiment progressed through each drought/recovery cycle, the initial difference in SWC between the CO_2_ treatments converged. This convergence of SWC between the CO_2_ treatments coincided with the mortality of grasses in the elevated CO_2_ mesocosms. We suggest that this is because there were fewer individuals (due to the extreme drought-related mortality) in the elevated CO_2_ mesocosms which would have reduced the total canopy transpiration and rainfall interception in those mesocosms.

We hypothesised that decreases in stomatal conductance under elevated CO_2_ would be greater for the C_4_ and native grasses. Surprisingly we found that the exotics had the greatest reduction in stomatal conductance among the grasses under elevated CO_2_. In contrast the C_4_ and native grasses had greater increases in LAI under elevated CO_2_ compared to the other grass species. It is often assumed that native plants are less influenced by water limited conditions (e.g. extreme drought) in comparison to exotic plants [22], [23]. Our findings suggest that the exotic grasses would be less influenced by extreme drought than C_4_ and native grasses in a high CO_2_ world. However it is difficult to suggest if this could significantly alter the species composition of grasslands. This is because changes in SWC can alter the structure and function of grasslands in aspects other than productivity that may influence species composition. SWC can contribute to species shifts within grasslands by changing seed production and seedling recruitment among species [6], [24] and altering competitive interactions among established plants [25], [26], [27].

The findings from our study suggest that CO_2_ concentration and soil water availability are important in mediating grassland productivity through grass dieback and mortality. It has been projected that a drastic shift in the annual global precipitation patterns may result in up to a 20% loss in soil water [28]. This is reinforced by global climate models which predict that large areas on every inhabitable continent will experience intensified droughts and widespread decreases in soil water [2]. However, net primary productivity in grasslands is projected to increase under elevated CO_2_ conditions. Parton et al [29] modelled the effects of increased CO_2_ for 31 temperate and tropical grassland sites, using the CENTURY model. They found that with the exception of cold desert steppe regions the net primary productivity of grasslands increased under elevated CO_2_ conditions [29]. From our results we suggest that this projected global increase in the productivity of grasslands under elevated CO_2_ levels may be negated (by grass dieback and mortality) due to the projected soil water constraints in the future, both as a direct consequence of changed precipitation and changes in plant-level traits such as stomatal conductance and LAI.

This study only examined the response of a single growth stage (i.e. mature) of the grasses to repeated extreme drought events under different CO_2_ treatments. However, the vulnerability of plants to climatic extremes may be different in the early stages of their growth and development in comparison to mature plants. Therefore it is important to consider also the effect of climatic extremes on seed germination and seedling establishment as these stages are likely to strongly influence vegetation dynamics [30].

Predicting responses at local and regional scales to global change is often dependent on scaling up from plant-level mechanisms [10]. Although we attempted to make our glasshouse based mesocosm experiment as realistic a representation of the field conditions as possible, it is almost an impossible exercise to exactly recreate these conditions. For example, the soil depth in our mesocosms was 28 cm which is shallow compared to soil depth in the field. This may have caused higher mortality of the grasses in the mesocosms as they did not have access to deeper soil water as grasses in field conditions may have. Mesocosm studies such as this are important for understanding mechanisms and stimulating further research, rather than simply assessing outcomes [31]. It is important to note that we did not try to quantify exact mortality of grasses in response to extreme drought, but rather we aimed to test a potential mechanism that may be important in determining mortality which can then be scaled up to a local and regional scale.

This study has shown that vegetation response to climatic extremes is likely to be affected by CO_2_ concentration, with extreme drought-related mortality of grasses increasing under elevated CO_2_ levels. The suggested mechanism for this increased mortality is an increase in the LAI of the grasses which offset reductions in stomatal conductance and increased rainfall interception prior to extreme drought events. This highlights the importance of considering the interactions between climatic extremes and other aspects of climate change such as CO_2_ concentration on plant communities. Our results also highlight the importance of better quantification of soil water in global climate and vegetation models as this may be a key driver affecting global vegetation patterns and responses to climate change.
